# Natural disease progression and novel survival prediction model for hepatocellular carcinoma with spinal metastases: a 10-year single-center study

**DOI:** 10.1186/s12957-020-01913-9

**Published:** 2020-06-20

**Authors:** Phichayut Phinyo, Chonmavadh Boonyanaruthee, Permsak Paholpak, Dumneoensun Pruksakorn, Areerak Phanphaisarn, Apiruk Sangsin

**Affiliations:** 1 Department of Family Medicine, Faculty of Medicine, Chiang Mai University, Chiang Mai, Thailand; 2 Center for Clinical Epidemiology and Clinical Statistics, Faculty of Medicine, Chiang Mai University, Chiang Mai, Thailand; 3 Department of Orthopedics, Faculty of Medicine, Chiang Mai University, Chiang Mai, Thailand; 4 Department of Orthopedics, Faculty of Medicine, Khon Kaen University, Khon Kaen, Thailand; 5 Musculoskeletal Science and Translational Research, Chiang Mai University, Chiang Mai, Thailand

**Keywords:** Decision making, Hepatocellular carcinoma, Prognosis, Prognostic factors, Spinal metastases, Survival analysis

## Abstract

**Background:**

Individual prediction of life expectancy in patients with spinal metastases from hepatocellular carcinoma (HCC) is key for optimal treatment selection, especially when identifying potential candidates for surgery. Most reported prognostic tools provide categorical predictions, and only a few include HCC-related factors. This study aimed to investigate the natural progression of the disease and develop a prognostic tool that is capable of providing individualized predictions.

**Methods:**

Patients with HCC-derived metastatic spinal disease were identified from a retrospective cohort of patients with spinal metastases who were diagnosed at Chiang Mai University Hospital between 2006 and 2015. Kaplain–Meier methods and log-rank tests were used to statistically evaluate potential factors. Significant predictors from the univariable analysis were included in the flexible parametric survival regression for the development of a prognostic prediction model.

**Results:**

Of the 1143 patients diagnosed with HCC, 69 (6%) had spinal metastases. The median survival time of patients with HCC after spinal metastases was 79 days. In the multivariable analysis, a total of 11 potential clinical predictors were included. After backward elimination, four final predictors remained: patients aged > 60 years, Karnofsky Performance Status, total bilirubin level, and multifocality of HCC. The model showed an acceptable discrimination at C-statistics 0.73 (95% confidence interval 0.68–0.79) and fair calibration.

**Conclusion:**

Four clinical parameters were used in the development of the individual survival prediction model for patients with HCC-derived spinal metastases of Chiang Mai University or HCC-SM CMU model. Prospective external validation studies in a larger population are required prior to the clinical implication of the model.

## Background

Hepatocellular carcinoma (HCC) is a rare type of cancer in Western countries; however, its prevalence in Eastern Asia and Southeast Asia is relatively high [1]. In Thailand, the age-standardized incidence rate of HCC was reported to be 22 per 100,000 person-years [2]. With improvements in diagnostic strategies, various anticancer therapies, and modern surgical and intervention techniques, the survival of HCC patients has increased by more than 2 years. Although HCC is not a highly osteophilic cancer, the prolongation of life expectancy of patients results in an increase in the incidence of bone metastasis [3]. Due to the highly vascularized nature of HCC, metastatic lesions in the bone cause severe pain and disability. Structural and functional disruption of the bone architecture increases the probability of pathologic fractures. In the case of spinal metastases, neurological deficits because of spinal cord compression may occur, which seriously deteriorates the patient’s quality of life [4].

Individual prognosis of survival after the diagnosis of spinal metastases is therefore crucial to aid clinicians to determine the optimal management for each specific HCC patient [5]. A life expectancy of more than 6 months is generally considered the ideal cutoff point for surgical management of the patient [6]. In the past, several series of prognostic factors and scoring systems were used in combination to predict patient survival. However, the prognostic factors incorporated within each score were heterogeneous [7], and several scores were derived from a cohort of only a few HCC patients [8–10]. Moreover, the clinical characteristics and median overall survival in each study were found to be different from those of the Thai HCC population. The appropriateness of implicating these prediction scores in the context of Thai population is questionable and might result in misclassification of patients.

In this study, we aimed to investigate and report on the natural disease progression in patients with HCC-derived spinal metastases in Thailand and identify the prognostic factors for survival in this patient cohort. Identified prognostic clinical parameters would be used to establish a novel, individualized survival prediction model for patients with HCC with spinal metastatic tumors.

## Methods

### Design, participants, and data collection

A prognostic prediction model was developed using a retrospective analysis of a large patient cohort. This study included 1143 patients with HCC who were diagnosed and managed at Chiang Mai University Hospital from 2006 to 2015. From the recruited patients, those who had HCC with spinal metastases were identified. All patient information was obtained from the Chiang Mai Cancer Registry. This study was approved by the institutional review board and ethical committee of the Faculty of Medicine, Chiang Mai University. Primary HCC was diagnosed based on either an official radiological report of computed tomography scan or tissue biopsy. Spinal metastases were diagnosed using one or more of the following modalities: pathologic report of a spinal biopsy, magnetic resonance imaging, computed tomography scan, nuclear bone scan, or positron emission tomography scan. Demographic data (e.g., gender, age), clinical characteristics (e.g., cirrhotic status, presence of ascites, hepatic encephalopathy, Child-Pugh classification, Karnofsky Performance Status (KPS; poor 20–40%, moderate 50–70%, and good 80–100%), and Frankel grade of preoperative neurological status (Complete Frankel A, B; Incomplete Frankel C, D; None Frankel E), tumor characteristics (e.g., data of primary tumor diagnosis, number of the primary tumor, tumor size, portal vein involvement, visceral organ metastasis, previous treatment received, date of skeletal and spinal metastases diagnosis, level of spinal metastases, number of vertebral column involved, and number of extraspinal bone metastases) were reviewed and obtained from the electronic medical record. All patients were assigned to a specific prognostic group according to both Tomita [8] and the revised version of the Tokuhashi score [10].

### Predictors

Candidate predictors were as follows: aged > 60 years, KPS, cirrhotic status, presence of ascites, total bilirubin level, serum albumin level, number of primary tumors, portal vein involvement, visceral organ metastasis, number of vertebral columns involved, and number of extraspinal bone metastases. The selection of predictors was based on the availability of predictors at the time of prediction, clinical expertise, and previously reported scoring system for prediction of metastatic spinal tumors, including Tomita [8] and revised Tokuhashi score [9, 10]. A total of 11 factors were included in univariate survival analysis using the Kaplan–Meier method for estimation of survival probabilities at 3, 6, and 12 months. Each predictor was categorized at a generally accepted cutoff point, according to those in the literature. The difference in survival distribution across prognostic covariates was examined using the log-rank test.

### Derivation of the survival model

All statistical analyses and model derivation procedures were carried out using Stata version 16 (StataCorp, TX, USA). A flexible parametric survival model, the Royston–Parmar (RP) model, was used to derive the prognostic model via the *stpm2* package. The main advantage of this non-rigid parametric survival model, which is beyond the Cox regression, is its ability to estimate baseline cumulative hazard function via the use of natural cubic splines. This allows for more accurate and precise individual predictions. Sensitivity analysis was used to select the appropriate scales and number of degrees of freedom for the baseline spline function. For our model, the cumulative hazard scale with four degrees of freedom was chosen based on the lowest Akaike information criterion (AIC) and Bayesian information criterion (BIC) values. Prior to fitting the RP model, the proportional hazard assumption was tested using Schoenfeld residuals, and any predictor that violated the assumption would be subsequently investigated for time-dependent effects using the *stpm2t* command. Eleven potential predictor variables were included in the multivariable flexible parametric model, *stpm2* command, with pre-specified scale and number of knots, as mentioned. Backward elimination of each predictor was carried out based on both a significant threshold of *P* value < 0.100 and likelihood ratio test.

### Multiple imputation

Three predictor variables were found to have more than 10% of the missing values, which could lead to biased survival estimates of the prognostic model if the complete-case analysis was performed. Multiple imputation with chained equation via *mi impute chained* command was used to generate missing values prior to model derivation. The number of imputed datasets was based on the highest percentage of incomplete variables, which was 15%. The logit model was chosen for the imputation of all three predictors (cirrhosis, ascites, and portal vein involvement). We included all potential predictor variables within the multivariable flexible parametric regression model via *mi estimate* commands and subsequently eliminated each predictor from the model via a backward elimination approach. The final predictors from both the imputed dataset and complete-case analysis were compared. A model with a higher discriminative ability was chosen for the final model derivation.

### Discrimination and calibration

The prognostic model performance was evaluated according to two main aspects. We evaluated the discriminative ability of the model to correctly distinguish a person with longer survival from a person with shorter survival via the use of Harrell’s C discrimination index (or C-statistics) for survival analysis. We also reported other measures of discrimination, such as Royston & Sauerbrei’s D statistic and ${R}_D^2$. The model calibration indicated the agreement between predicted survival probabilities and observed proportions of survival outcomes. We examined the calibration of the derived model via calibration plots. Both predicted risks and observed outcomes were separated into five equally distributed quantiles. The calibration was evaluated based on the inspection of agreement between the model-predicted survival curve and Kaplan–Meier survival curve within each quantile.

### Internal validation

The bootstrap resampling method was used for assessing model optimism and internal validation. Two hundred samples were randomly sampled, having the same size as the original dataset with the replacement of the sampled record. The entire modeling process was performed in each bootstrap sample to yield a final of 200 bootstrap models. Harrell’s C-statistics were calculated and averaged for all derived models. Then, each bootstrap model was subsequently applied to the original dataset. The average of Harrell’s C-statistics was again estimated. The model optimism of Harrell’s C-statistics was calculated by subtracting two averaged Harrell’s C-statistics. We also reported the optimism of other measures of discrimination, such as Royston & Sauerbrei’s D statistic, ${R}_D^2$, and the shrinkage factor for external validation studies.

### Model presentation

Each patient’s predicted survival probabilities at each clinically relevant time point (3, 6, and 12 months) from the flexible parametric model were classified into five risk groups with specific coloring label as follows: 81–100% (green), 61–80% (yellow), 41–60% (light orange), 21–40% (dark orange), and 0–20% (pink). The prognostic model was presented as a score chart for simplicity and applicability. The cross-tabulate score chart comprised up to four final predictors. The estimated survival probabilities and their 95% confidence intervals were presented within each cell of the chart. Based on the point estimate of estimated survival probability, each cell was colored according to the five pre-specified risk groups.

### Comparative validation with the conventional scoring system

Both the Tomita score and the revised Tokuhashi score were calculated for each patient within the cohort from the available data. Assuming that the appropriate cutoff point for operative management was a score-predicted survival of more than 6 months, we estimated the sensitivity, specificity, and area under the receiver operating characteristics curve (AuROC) from each model by comparing the predicted survival status at 6 months with the observed survival endpoints at 6 months for each patient. We then compared the diagnostic performance of both conventional scores with our prediction based on the score chart where patients whose 6-month survival probabilities lied within the green and yellow groups; those with more than 60% chance of survival were considered as proper candidates for surgical management.

## Results

### Patient characteristics

From 2006 to 2015, a total of 1143 HCC patients were identified, of which 69 (6%) had HCC with spinal metastases accounting for 5.8% of all spinal metastasis patients treated in our hospital. Most of the patients were non-elderly men with moderate to poor KPS.

HCC and spinal metastases were simultaneously diagnosed in 24 (34.8%) patients. The remaining 45 (65.2%) patients developed spinal metastases 30–1074 days after HCC diagnosis, with a median duration of 196 days. Thirty-seven patients were hepatitis B virus (53.6%) and 16 (23.2%) were hepatitis C positive, while the remaining 16 (23.2%) had a history of alcoholic cirrhosis. Two or more spinal metastases were observed in 30 (43.5%) patients. The area of the affected spine included the combined region of the affected spine (31.8%), thoracic (36.3%), lumbar (24.6%), and cervical (7.3%). The most common skeletal-related events (SRE) were neural compression (33.3%), pain (27.5%), and pathologic fracture (8.7%). A total of 30.5% of spinal metastases were detected incidentally during the workup of the primary tumor and tumor staging. For primary HCC treatment, five (7.2%) patients underwent primary tumor resection and 15 (21.7%) underwent palliative intervention, such as percutaneous ethanol injection, transcatheter chemoembolization, and/or radiofrequency ablation, while 49 (71.1%) received best supportive care. For the treatment of spinal metastasis, two (2.9%) patients received palliative spinal surgery with postoperative external beam radiation (EBRT), 35 (50.7%) received EBRT alone, and 32 (46.4%) received palliative treatment. The clinical characteristics are summarized in Table 1.

**Table 1:** Clinical characteristics of the patient cohort

Clinical Characteristics	Total, *n* (%)	Missing, *n* (%)	Hazard ratio* (95% CI)	3-month survival (%)	6-month survival (%)	12-month survival (%)	*P* value**
Gender							
Male	60 (87.0)	0 (0)	Reference	45.0	33.3	16.7	0.520
Female	9 (13.0)		0.79 (0.39, 1.61)	66.7	44.4	22.2	
Age group (years)							
≤ 60 years	50 (72.5)	0 (0)	Reference	52.0	38.0	22.0	0.036
> 60 years	19 (27.5)		1.78 (1.03, 3.08)	36.8	26.3	5.3	
Karnofskys Performance Status							
Good	17 (24.7)	0 (0)	Reference	82.4	70.6	41.2	0.003
Moderate	25 (36.2)		2.29 (1.15, 4.54)	48.0	28.0	12.0	
Poor	27 (39.1)		3.16 (1.63, 6.15)	25.9	18.5	7.4	
Cirrhosis							
No	21 (30.4)	7 (10.2)	Reference	66.7	47.6	23.8	0.024
Yes	41 (59.4)		1.95 (1.08, 3.51)	39.0	29.3	17.1	
Ascites							
No	33 (47.8)	10 (14.5)	Reference	66.7	51.5	27.3	0.001
Yes	26 (37.7)		2.44 (1.39, 4.28)	26.9	15.4	7.7	
Total bilirubin (mg/dL)							
< 2.0	55 (79.7)	1 (1.5)	Reference	56.4	43.6	21.8	< 0.001
2.0–3.0	5 (7.2)		2.75 (1.06, 7.15)	20.0	0	0	
> 3.0	8 (11.6)		11.25 (4.39, 28.86)	0	0	0	
Serum albumin (mg/dL)							
> 3.5	25 (36.2)	1 (1.5)	Reference	68.0	56.0	24.0	0.056
2.8–3.5	26 (37.7)		1.38 (0.78, 2.45)	30.8	23.1	19.2	
< 2.8	17 (24.6)		2.17 (1.14, 4.14)	41.2	23.5	5.9	
Number of primary tumor							
Single tumor	14 (20.3)	2 (2.9)	Reference	71.4	50.0	35.7	0.018
Multiple tumors	53 (76.8)		2.20 (1.13, 4.29)	43.4	32.1	13.2	
Portal vein involvement							
No	28 (40.6)	11 (15.9)	Reference	71.4	50.0	25.0	0.196
Yes	30 (43.5)		1.43 (0.83, 2.45)	33.3	26.7	16.7	
Visceral organ metastasis							
No	35 (50.7)	2 (2.9)	Reference	65.7	51.4	25.7	0.017
Yes	32 (46.4)		1.35 (1.05, 1.73)	31.3	18.8	9.4	
Number of vertebral columns involved							
1	30 (43.5)	3 (4.4)	Reference	53.3	40.0	20.0	0.751
2	19 (27.5)		0.95 (0.52, 1.73)	57.9	36.8	15.8	
≥ 3	17 (24.6)		1.21 (0.66, 2.22)	35.3	29.4	17.7	
Number of extra spinal bone metastases							
0	32 (46.4)	7 (10.2)	Reference	59.4	43.8	25.0	0.256
1–2	21 (30.4)		1.52 (0.86, 2.69)	42.9	33.3	14.3	
≥ 3	9 (13.0)		1.64 (0.74, 3.63)	33.3	22.2	11.1	

Abbreviations: *CI* confidence interval

*Hazard ratio from univariable Cox’s proportional hazard regression

**P value from log-rank test

### Survival rate of patients with HCC with spinal metastases

The median survival time of the cohort was 79 days (95% CI, 62–118 days) with the longest duration of follow-up for a single patient at 930 days. At the end of the study, only two (2.90%) patients had censored observations. The overall survival rates for HCC with spinal metastases at 3, 6, 9, 12, and 24 months were 47.8%, 34.8%, 24.6%, 17.4%, and 1.8%, respectively.

### Candidate predictors

**Fig. 1:**
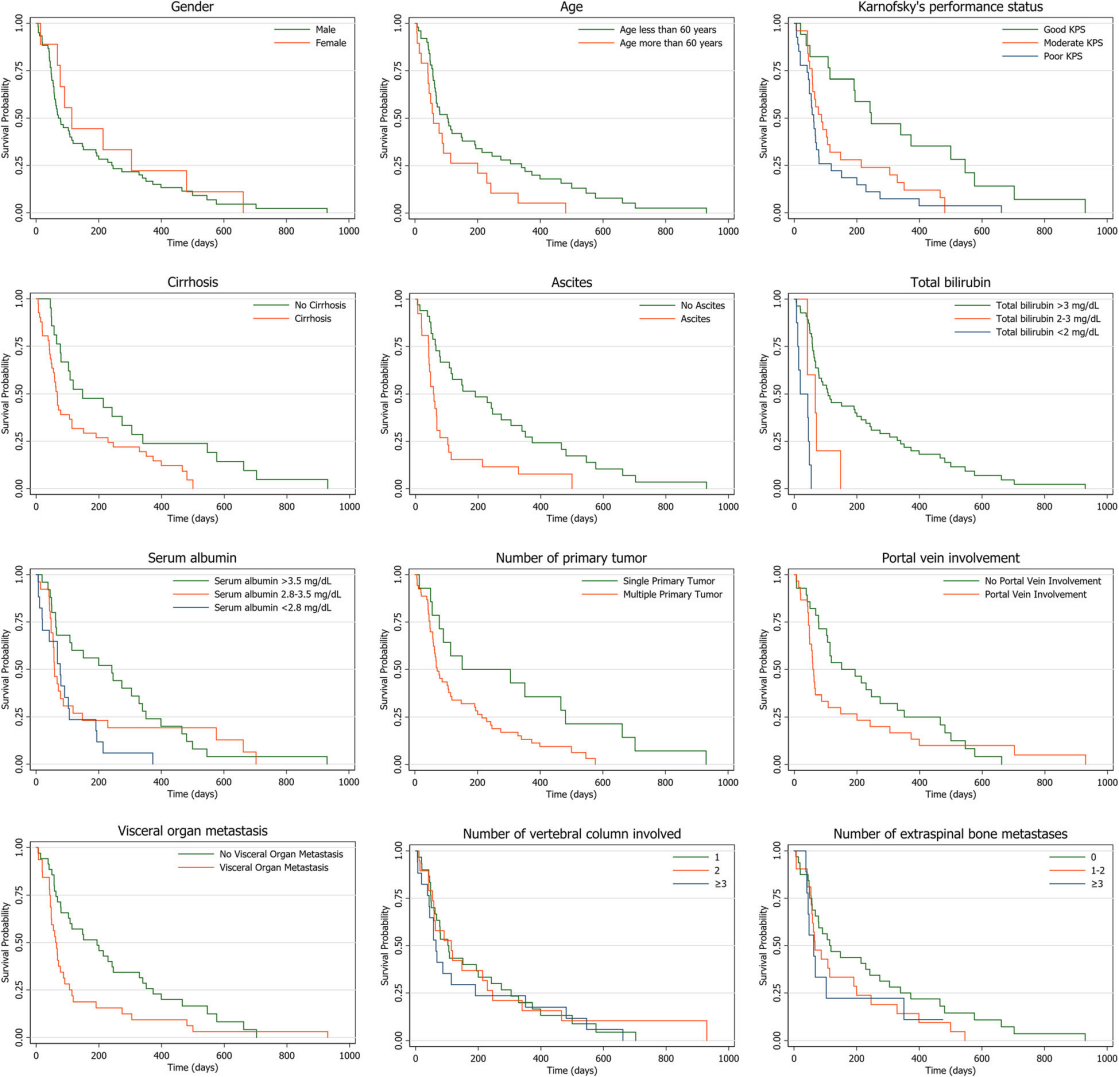
Kaplan–Meier curves visualizing differences in survival distribution among patients with and without prognostic factors

From the univariable log-rank analysis, seven clinical characteristics were identified as potential predictors of survival for HCC patients with spinal metastasis: aged > 60 years (*P* = 0.036), moderate and poor Karnofskys Performance Status (*P* = 0.003), the presence of cirrhosis (*P* = 0.024), the presence of ascites (*P* = 0.001), total bilirubin level ≥ 2 mg/dL (*P* < 0.001), HCC with multifocal tumors (*P* = 0.018), and the presence of visceral organ metastasis (*P* = 0.017). The survival probabilities at 3, 6, and 12 months for each predictor were estimated and depicted by Kaplan–Meier curves (Fig. 1).

### Final predictors

All candidate predictors listed in Table 1 were included in the full multivariable prediction model via flexible parametric survival regression, regardless of their statistical significance from univariable analyses. No statistical evidence of violation of proportional hazard assumption was found in the Schoenfeld residuals test (*P* = 0.944). To reduce the number of predictors, backward elimination was performed based on a critical *P* value < 0.1 and on the likelihood ratio test of each model after the elimination of non-significant predictors. The modeling procedures were performed with both the multiple imputed method and complete-case analysis, and the results of each model were compared. In this study, both models yielded the same final covariates within the model; therefore, a model based on complete-case analysis was reported.

Four final predictors remained within the reduced model: aged > 60 years (hazard ratio [HR] 1.77, 95% confidence interval [CI] 0.97–3.23, *P* = 0.062), moderate and poor KPS (HR 2.00, 95% CI 0.96–4.18, *P* = 0.066 and HR 2.96, 95% CI 1.48–5.92, *P* = 0.002, respectively), total bilirubin level ≥ 2 and ≥ 3 mg/dL (HR 2.22, 95% CI 0.82–5.99, *P* = 0.114 and HR 10.4, 95% CI 3.92–27.82, *P* < 0.001, respectively), and multiple foci of HCC (HR 2.63, 95% CI 1.29–5.35, *P* = 0.008) (Table 2). The estimated beta-coefficients for all predictor variables on the hazard scale and their 95% CIs from both the full and reduced models are shown, see Supplementary Table 1, Additional file 1. Predictors with a positive beta-coefficient increased the probability of mortality, whereas predictors with a negative beta-coefficient decreased the probability of mortality. The reported beta-coefficients could be converted to hazard ratios by exponentiation of the beta-coefficients.

**Table 2:** Estimated hazard ratios in the full and reduced multivariable flexible parametric regression models

Predictors	Full model			Reduced model		
	HR	95% CI	*P* value	HR	95% CI	*P* value
Age group (years)						
≤ 60	1.00	Reference		1.00	Reference	
> 60	2.13	0.99, 4.54	0.052	1.77	0.97, 3.23	0.062
Karnofskys Performance Status						
Good	1.00	Reference		1.00	Reference	
Moderate	1.86	0.71, 4.88	0.204	2.00	0.96, 4.18	0.066
Poor	3.64	1.61, 8.21	0.002	2.96	1.48, 5.92	0.002
Cirrhosis						
No	1.00	Reference			Not included	
Yes	1.20	0.60, 2.39	0.600			
Ascites						
No	1.00	Reference			Not included	
Yes	1.05	0.32, 3.42	0.935			
Total bilirubin (mg/dL)						
< 2.0	1.00	Reference		1.00	Reference	
2.0–3.0	3.27	0.20, 12.59	0.085	2.22	0.82, 5.99	0.114
> 3.0	9.22	2.46, 34.50	0.001	10.44	3.92, 27.82	< 0.001
Serum albumin (mg/dL)						
> 3.5	1.00	Reference			Not included	
2.8–3.5	0.80	0.34, 1.91	0.619			
< 2.8	2.66	1.05, 6.71	0.039			
Number of primary tumor						
Single tumor	1.00	Reference		1.00	Reference	
Multiple tumor	3.42	1.23, 9.51	0.019	2.63	1.29, 5.35	0.008
Portal vein involvement						
No	1.00	Reference			Not included	
Yes	1.46	0.75, 2.85	0.265			
Visceral organ metastasis						
No	1.00	Reference			Not included	
Yes	1.19	0.85, 1.67	0.301			
Number of vertebral columns involved						
1	1.00	Reference			Not included	
2	1.10	0.52, 2.32	0.807			
≥ 3	1.70	0.69, 4.16	0.248			
Number of extraspinal bone metastases						
0	1.00	Reference			Not included	
1–2	0.93	0.31, 2.78	0.892			
≥ 3	1.03	0.33, 3.19	0.964			

Abbreviations: *HR* hazard ratio, *CI* confidence interval

### Model calibration and discrimination

**Fig. 2:**
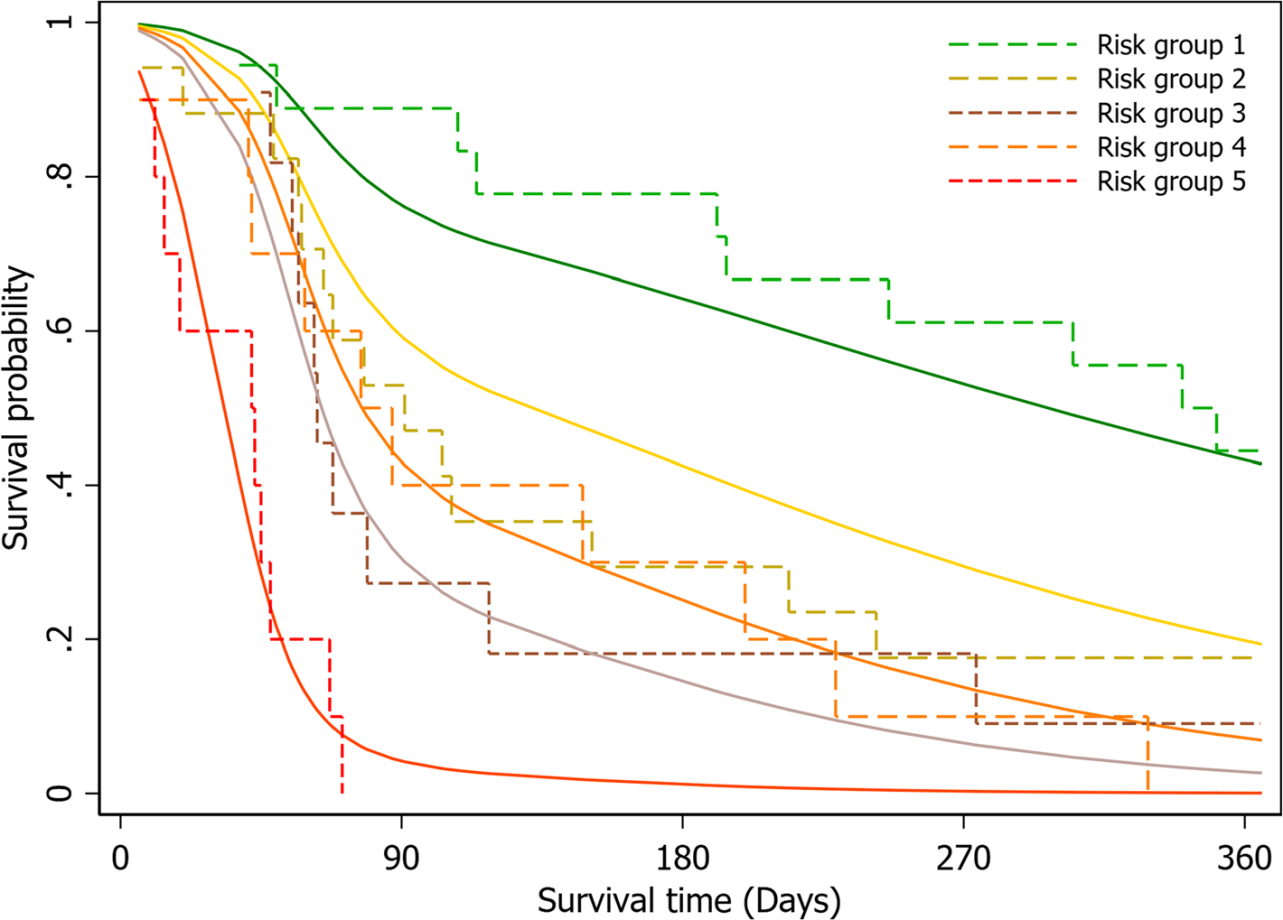
Calibration plots compare the model-predicted risk and the observed outcomes against one another within each of the risk quantiles

For the measure of discrimination, the Harrell C-statistics for the full and reduced models were 0.77 and 0.72, respectively. The Somers’ D was 0.55 for the full model and 0.44 for the reduced model. The Royston & Sauerbrei’s D statistic and ${R}_D^2$ of the reduced model were 1.571 (SE 0.274) and 0.37 (95% CI 0.20–0.51), respectively. The calibration of the final model was visualized with a calibration plot, where predicted risk and observed outcomes were compared against one another within each of the different risk quantiles (Fig. 2). The prognostic model appeared to be well calibrated in the third, fourth, and fifth risk quantiles. The model overestimated the probability of death in the first risk quantile and underestimated the probability of death in the second risk quantile.

### Internal validation

Internal validation of the derived prognostic model was performed via a bootstrap resampling procedure with 200 replicates. The apparent C-statistics and the test C-statistics were 0.73 (95% CI 0.68–0.79, min 0.61, max 0.81) and 0.71 (95% CI 0.70–0.71, min 0.64, max 0.73), respectively. The C-statistic optimism was 0.023 (95% CI, 0.017–0.028). The Royston & Sauerbrei’s D statistic optimism was 0.31 (apparent D 1.79 and test D 1.48), and the ${R}_D^2$ optimism was 0.09 (apparent ${R}_D^2$ 0.43 and test ${R}_D^2$0.34). The shrinkage factor was 0.82 (95% CI 0.80–0.84). The regression coefficients in subsequent validation studies should be multiplied by this factor to yield a more reliable estimate of the predicted probabilities.

### Demonstration of individual predictions from the HCC-SM CMU model

**Fig. 3:**
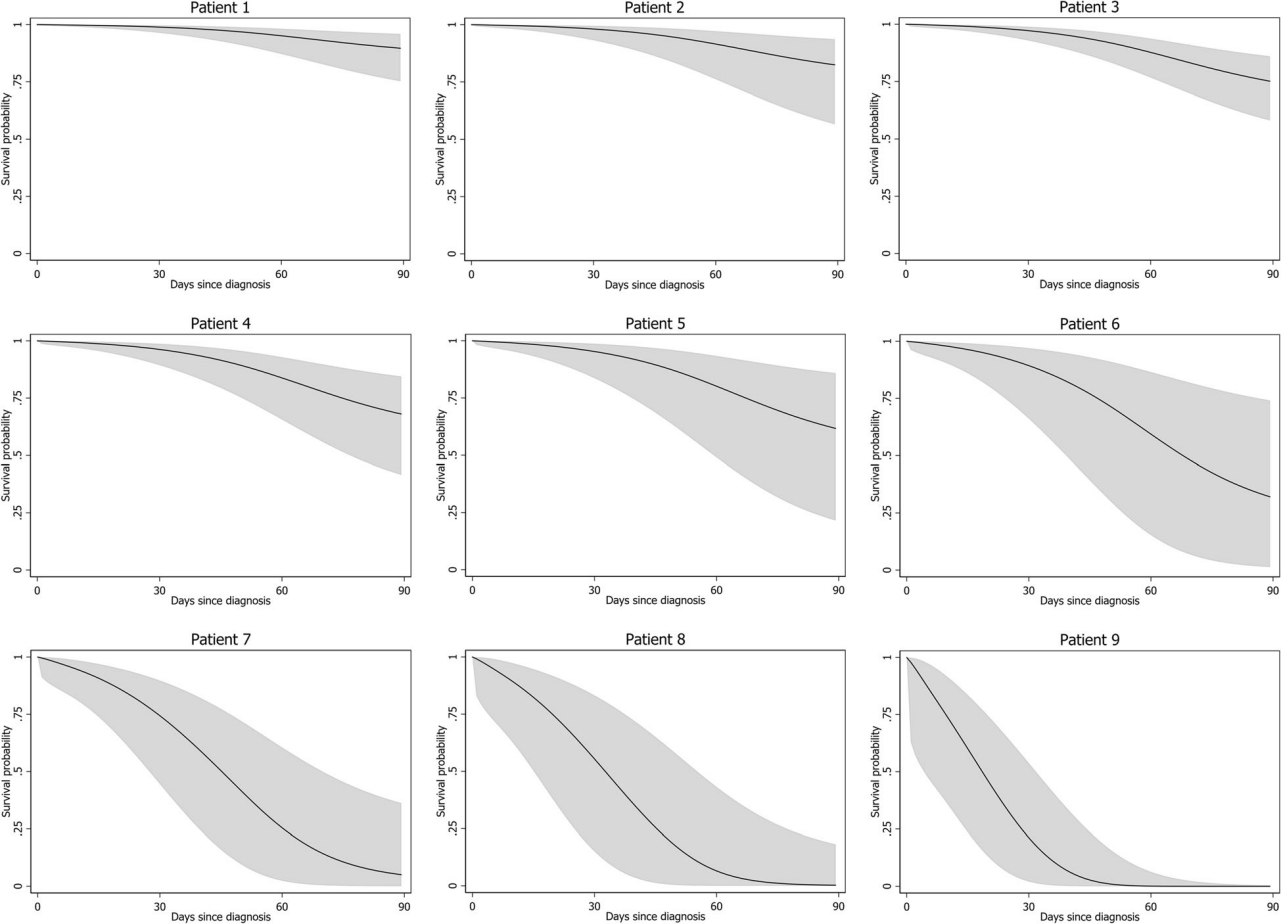
Individual prediction curves of nine randomly selected patients from the study cohort

**Fig. 4:**
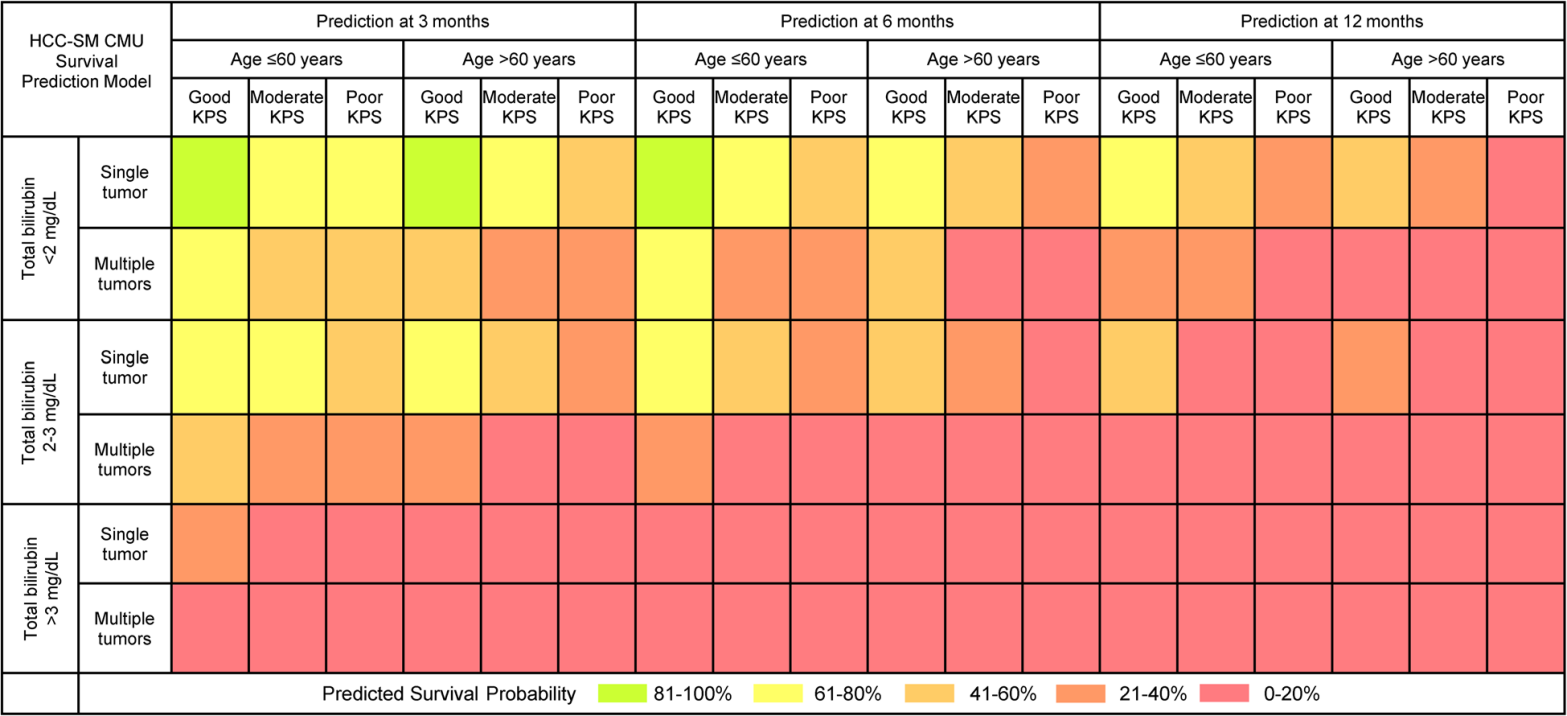
HCC-SM CMU score chart. Each patient’s predicted survival probabilities at each clinically relevant time point (3, 6, and 12 months) classified into five risk groups with a specific coloring label as follows: 81–100% (green), 61–80% (yellow), 41–60% (light orange), 21–40% (dark orange), and 0–20% (pink). Abbreviation: KPS, Karnofsky Performance Status

From the final remaining predictors of the newly derived HCC with spinal metastasis model by Chiang Mai University or HCC-SM CMU model, all HCC patients with spinal metastasis were individually categorized into 36 possible subcategories. The survival probabilities of each patient were estimated at three specific time points (3, 6, and 12 months) based on the HCC-SM CMU model. Each of the 36 subtypes of HCC patients was assigned a specific survival probability with a 95% CI (Supplementary Tables 2, 3, and 4, Additional file 1). For demonstration purposes, we selected nine possible types of patients with different combinations of predictor variables and presented, along with patient profile, the model estimated survival probabilities of each patient at 3, 6, and 12 months (Table 3). The individual prediction curves of each patient are presented in Fig. 3. For clinical applicability, the HCC-SM CMU score chart, where four predictors were cross-tabulated and each cell was labeled with survival probability and colored according to pre-specified risk groups was introduced (Fig. 4).

**Table 3:** Demonstration of the model-estimated survival probability at each time point from nine sample patients

Input predictor variables					Model estimation of survival probability (%, 95% confidence interval)		
No	Age	KPS	Total bilirubin	Number of primary tumor	3 months	6 months	12 months
1	41	Good	0.8	Single	89.5 (74.9–95.9)	83.4 (62.1–93.3)	69.4 (43.1–85.4)
2	64	Good	1.2	Single	82.2 (56.2–93.6)	72.5 (38.9–89.6)	52.4 (17.8–78.5)
3	52	Good	1.8	Multiple	74.8 (57.6–85.8)	62.0 (41.2–77.3)	38.3 (19.4–57.1)
4	66	Moderate	1.6	Single	67.6 (41.0–84.2)	52.5 (23.3–75.2)	27.5 (6.5–54.4)
5	56	Moderate	2.3	Single	61.2 (21.0–85.7)	44.6 (7.1–78.2)	19.8 (0.6–60.0)
6	46	Good	3.2	Single	31.5 (1.2–73.8)	15.0 (0.1–62.2)	2.2 (0–35.9)
7	48	Good	3.4	Multiple	4.8 (0–35.7)	0.7 (0–19.9)	0 (0–3.6)
8	69	Poor	3.8	Single	0.2 (0–17.7)	0 (0–6.6)	0 (0–0.4)
9	76	Poor	6.9	Multiple	0 (0–0.4)	0 (0)	0 (0)

Abbreviation: *KPS* Karnofsky Performance Status

### Comparative validation of the HCC-SM CMU model

We performed a comparative validation of the HCC-SM model with classical scoring systems such as the Tomita score and the revised Tokuhashi score. This analysis could be done in 58 (84.1%) patients within the cohort, due to incomplete data on the component of the revised Tokuhashi score in 11 patients. By deciding to perform surgical management only in patients whose predicted survival probability at 6 months was more than 80% (only the green risk group), the HCC-SM CMU model would provide clinicians with 100% specificity or absence of false-positive cases (predicted to be alive at 6 months, but actually died before the time). However, this approach would result in higher false-negative rates where patients who survived beyond 6 months were predicted to be dead before 6 months and thus missed the opportunity to undergo surgery. In contrast, if patients whose predicted risk fell within either the green or yellow group at 6 months were chosen for surgical treatment, the HCC-SM CMU model would provide higher sensitivity at 56.5% and 88.6% specificity. In this case, a smaller number of patients would be misclassified, and more patients would be appropriately managed. We estimated the sensitivity, specificity, positive predictive value, negative predictive value, and AuROC of the Tomita score and the revised Tokuhashi score assumed that they had been used to categorize patients for operation in this cohort of patients (Table 4). The Kaplan–Meier curves showing the difference in survival distribution of each score in differentiation of patients who would survive more or less than 6 months are depicted, see Supplementary Figure 1, Additional file 1.

**Table 4:** Comparative validation of the HCC-SM CMU survival prediction model with conventional scoring systems

	Status at 6 months			Sensitivity % (95% CI)	Specificity % (95% CI)	PPV % (95% CI)	NPV % (95% CI)	AuROC (95% CI)
	Survived	Died	Total					
HCC-SM CMU prediction model (only green group at 6 months)								
Alive	2	0	2	8.7 (1.1–28.0)	100.0 (90.0–100.0)	100.0 (15.8–100.0)	62.5 (48.5–75.1)	0.54 (0.48–0.60)
Not alive	21	35	56					
	23	35	58					
HCC-SM CMU prediction model (green and yellow group at 6 months)								
Alive	13	4	17	56.5 (34.5–76.8)	88.6 (73.3–96.8)	76.5 (50.1–93.2)	75.6 (59.7–87.6)	0.73 (0.61–0.84)
Not alive	10	31	41					
	23	35	58					
Tomita score^a^								
Alive	17	15	32	73.9 (51.6–89.8)	57.1 (39.4–73.7)	53.1 (34.7–70.9)	76.9 (56.4–91.0)	0.66 (0.53–0.78)
Not alive	6	20	26					
	23	35	58					
Revised Tokuhashi score^b^								
Alive	8	5	13	34.8 (16.4–57.3)	85.7 (69.7–95.2)	61.5 (31.6–86.1)	66.7 (51.0–80.0)	0.60 (0.49–0.72)
Not alive	15	30	45					
	23	35	58					

Abbreviations: *AuROC* area under receiver operating characteristics curve, *CI* confidence interval, *PPV* positive predictive value, *NPV* negative predictive value

^a^The Tomita score predicts the patients to be alive at 6 months if the score is 2–7 points. Patients with a Tomita score of > 8 points are predicted not to be alive at 6 months

^b^The Revised Tokuhashi score predicts for the patient to be alive at 6 months if the score is 9–15 points. Patients with a Tomita score of < 9 points are predicted not to be alive at 6 months

## Discussion

In this study, we have summarized the natural disease progression and survivals of patients with HCC-derived spinal metastases and explored for potential prognostic factors for prediction of survival to derive a novel individualized prediction tool.

The incidence of HCC in Southeast Asian countries, including Thailand, is high [11]. Hepatitis B and C virus infection and alcoholic cirrhosis were important etiologies in our patients, which differed from a 36 patients case series from China, which were all derived from hepatitis B virus infection [5]. From our 10-year study, only 6% of patients with HCC were found to have spinal metastases with a median survival time of 79 days, which was relatively short compared with that of other common metastatic tumors to the spine such as lung cancer (11.3 months) [12], breast cancer (21.7 months) [13], prostate cancer (58.3 months for hormone naïve and 5 months for hormone refractory [14, 15]), thyroid cancer (15.4 months) [16], and cholangiocarcinoma (3 months) [17]. In comparison with other reports on the survival of patients with HCC-derived spinal metastasis [6, 18, 19], the median survival time after metastasis was also shorter in our study. This could be explained by the lower rate of primary surgical resection and higher rate of palliative treatment and best supportive care in our study. As the median survival time after the diagnosis of spinal metastases was much shorter than the median time from diagnosis of primary HCC to the diagnosis of spinal metastases, the existence of undetectable occult spinal metastases was plausible.

Several studies have reported a variety of prognostic factors for survival in patients with HCC with spinal metastases. Interestingly, most of these factors were either patient-related (i.e., Eastern Cooperative Oncology Group or ECOG and KPS) [6, 20–22], liver-related (i.e., serum albumin level, serum lactate dehydrogenase or LDH, and Child-Pugh classification) [6, 21, 23, 24], or metastatic-related (visceral metastasis, other extrahepatic metastasis other than bone metastases) characteristics [22–25]. For tumor-related or intervention-related factors, primary HCC control or response to HCC treatment (i.e., response to radiotherapy, previous resection of primary HCC) were reported to be associated with patient survival after the diagnosis of spinal metastases [6, 20, 22, 24]. Previous scoring systems (i.e., Tomita score and Revised Tokuhashi score) have also been reported to be capable of survival prediction in this domain of patients [5, 23, 26]. However, other potentially important HCC-related factors such as the number of tumors (or multifocality) and tumor size have never been investigated as prognostic factors in patients with spinal metastasis. In this study, we included the number of tumors as a candidate predictor, as it was significantly associated with both recurrence and metastasis in HCC, even after adequate primary HCC control [27]. In contrast, the tumor size was not modeled as only a few patients (14.5%) had tumor size less than 5 cm, and the survival distribution between groups with tumor size smaller or larger than 5 cm was not significantly different. To allow for comprehensive and accurate survival prediction, our model incorporated all relevant aspects of the cancer, which were patient-, liver-, and tumor-related factors.

For patient-related factors, age and KPS were identified as significant prognostic factors in our study and were included as predictors in the model. Advanced age has been widely reported to be associated with poor survival outcomes in HCC, regardless of the staging or therapeutic modes [28, 29], but has never been explored in patients with HCC with spinal metastases. Previous studies used a variety of age cut points, ranging from 60 to 70 years old [27–30]. Our study is the first one to demonstrate the effect of old age (> 60 years) on the survival of patients with HCC-derived spinal metastases. The patient’s performance status, either via ECOG or KPS, was consistently identified as an influential survival factor in HCC patients with spinal metastases [6, 23] and were included as predictors in two recently developed prediction models: HCC-SM GPA by Rim et al. [24] and another scoring system by Uei and Tokuhashi [6].

For liver-related factors, only serum total bilirubin was found to be an independent survival predictor in our patient cohort. Serum bilirubin had never been independently explored as a prognostic factor in HCC-derived spinal metastasis, as all previous studies only examined the effect of serum albumin level or Child-Pugh classification as a whole [6, 21, 23, 24]. In our analysis, we separated the component of Child-Pugh classification to examine their independent effect on patient survival. Both serum albumin and serum bilirubin were found to be significant predictors in the multivariable model, but only serum bilirubin remained in the reduced model, which could be explained by the limited study size and that the effect estimates of serum bilirubin were much larger. Commonly, hyperbilirubinemia is a dominant marker of liver damage or liver failur e[31]. It was recently found that elevated serum bilirubin (≥ 1.5 mg/dL) was associated with HCC aggressiveness, increased risk of portal vein thrombosis (PVT), and lower survival, regardless of the tumor size. Moreover, patients with hyperbilirubinemia were found to have lower platelet counts, lower serum albumin, higher aspartate aminotransferase (AST), and higher alkaline phosphatase (ALP) levels than patients with normal bilirubin levels [32]. In our data, both serum albumin level and PVT revealed significant trends across the ordered groups of total bilirubin (*P* value from non-parametric test for trend 0.021 and 0.035, respectively).

For tumor-related factors, the multifocality of HCC was revealed to be another independent prognostic factor for survival in HCC-derived spinal metastasis. Multiple primary tumors reflect intrahepatic metastasis. Even when visible tumors are adequately resected, the remaining small metastases may still lead to recurrence and metastasis of HCC [33]. Metastatic-related factors such as visceral organ metastasis or metastases to the major internal organ, number of extraspinal bone metastases, and number of vertebral columns involved in spinal metastasis were not identified as significant predictors in our study, which was in concordance with other studies [6, 7]. Due to the limitations in terms of study sample size and incomplete data availability, we did not include intervention-related factors such as primary HCC control modality, treatment with bone-modifying agent, and sorafenib as predictors. In addition, more than 2/3 of the patients in this study (68.1%) did not receive definite HCC treatment and only three patients (4.4%) were offered surgical resection, and most patients received best supportive care or palliative treatment. It was quite evident from our exploratory analysis that prognostic factors for HCC survival at diagnosis of the cancer still affected the patient’s life expectancy after spinal metastases occurrence.

Most clinical decision tools for survival prediction in patients with metastatic spinal tumors were developed using the conventional Cox’s proportional hazard regression model because of its statistical simplicity and comprehensible concept [6, 8, 9, 34]. However, as a semi-parametric method, the baseline hazard function cannot be directly estimated from the model itself without conditioning the estimated regression coefficients, and the rigid proportional hazard assumption must be fulfilled for valid estimates. In addition, the model was specifically designed to assess the effect of each prognostic covariate on the change in the patient’s hazard function; it was not intended to predict the survival function of each patient [35]. For derivation of Cox’s prognostic model, weighing of coefficients was generally performed to generate a score for each patient from a combination of predictors. The score was subsequently categorized into different risk groups at arbitrary cutoff points for clinical application. Thus, precise individual prediction could not be performed using the Cox’s model [36].

In this study, we employed an alternative approach to directly estimate both the baseline hazard function and the individual survival function via the RP flexible parametric survival model. The RP model has been proven to yield more accurate predictions and more precise calibration in survival prediction [36, 37]. In deciding the appropriate treatment plan for HCC patients with spinal metastasis, an accurate survival prediction at clinically relevant time points is needed for each specific patient, especially for surgical management (either excisional or palliative). Applying predictions based on categorized risk groups might be considered unsophisticated as the splitting of an initially wide range of continuous prediction results in significant loss of information and certainly impairs the accuracy of overall prognostication [36]. With the HCC-SM CMU model, we proposed a novel practical tool for individualized prediction of survival probabilities in HCC patients with spinal metastases. The HCC-SM CMU model incorporates four significant clinical predictors to approximate survival probabilities and their confidence intervals for a specific patient at multiple time points.

In comparison to the widely used Tomita and Revised Tokuhashi score, our model carried higher discriminative ability in terms of C-statistics, as the other scores were not derived from the full cohort of patients with HCC-derived spinal metastasis. With four simple and readily available predictors, the application of the HCC-SM CMU model in practice would result in lower false-positive cases than both the Tomita and Revised Tokuhashi scores. As the model was intended to be used for guiding the need for major surgical operations, a higher specificity was indeed more important than the sensitivity. However, all differences in diagnostic indices were not statistically evident because of the limited statistical power. The most outstanding point of the HCC-SM CMU model over the traditional scores, including the HCC-SM GPA and the newly derived prognostic scoring system by Uei and Tokuhashi, was that it allowed for a wider range of predictions as seen from the score chart. For each clinically relevant time point, the HCC-SM CMU model exhibited 36 predicted survival probability values according to the individual characteristic pattern. These individual predictions could play an important role in risk communication and decision making from both the patient and physician perspectives [38].

Our study has several limitations. First, the model was derived from a small-sized patient cohort with retrospective data collection. Even though the number of censored observations was minimal, it is questionable whether the available number of events and total follow-up duration would be sufficient for model derivation with a flexible parametric model, which requires more parameters for the natural cubic spline function. Thus, to prevent model overfitting and optimism, we limited the number of final predictors and used a bootstrap resampling procedure to assess the presence of optimism and generate the shrinkage factor for further validation [39]. In terms of missing data, both the multiple imputation method and complete-case analysis were used to estimate the multivariable prediction model. Second, all predictors were modeled as categorical variables instead of continuous variables. This could result in the loss of information and an overoptimistic model in case data-driven approaches have been used. In our study, all predictors were categorized according to prior clinical scoring systems and generally accepted cut points to prevent optimistic model performance [40, 41]. Third, the model calibration was poor in specific ranges of predicted probabilities, and the discriminative ability was only fair to acceptable. Regarding calibration, careful interpretation of predicted survival probabilities was suggested, especially in the second risk quantile, where the survival probabilities were overestimated. In contrast, patients with predicted probabilities above 60% at 180 days would have an even higher chance of surviving beyond 6 months and therefore be a good candidate for surgical management. Prior to the clinical implication of the HCC-SM CMU model, a prospective external validation study with a larger HCC population is warranted.

## Conclusions

This study provided insight into the natural history of disease progression, magnitude of the disease, and prognostic factors for survival in patients with HCC-derived spinal metastases in Thailand. A novel survival prediction tool was also developed, which can support physicians for decision making in the optimal management of patients with spinal metastasis by considering surgical treatment only when patients are likely to live long enough to get benefit.

## Supplementary information

**Additional file 1: Supplementary Table 1.** Estimated log hazard ratios in the full and reduced multivariable flexible parametric regression models. **Supplementary Table 2.** Score chart with predicted survival probabilities at 3 months after diagnosis of spinal metastases. **Supplementary Table 3.** Score chart with predicted survival probabilities at 6 months after the diagnosis of spinal metastases. **Supplementary Table 4.** Score chart with predicted survival probabilities at 12 months after the diagnosis of spinal metastases. **Supplementary Figure 1.** Kaplan–Meier curves for comparative validation between the newly derived model’s prediction and the traditional Tomita and Revised Tokuhashi’s score in predicting which patients would survive more or less than 6 months. The *P*-values of the log-rank test are shown for each panel.

## Data Availability

The datasets used and/or analyzed during the current study are available from the corresponding author on reasonable request.

## Abbreviations

AIC: Akaike information criterion
ALP: Alkaline phosphatase
AST: Aspartate aminotransferase
AuROC: Area under receiver operating characteristics curve
BIC: Bayesian information criterion
CI: Confidence interval
CMU: Chiang Mai University
EBRT: External beam radiation therapy
ECOG: Eastern Cooperative Oncology Group
HCC: Hepatocellular carcinoma
HCC-SM CMU: Individual survival prediction model for patients with HCC-derived spinal metastases of Chiang Mai University
HR: Hazard ratio
KPS: Karnofsky Performance Status
LDH: Lactate dehydrogenase
PVT: Portal vein thrombosis
RP: Royston-Parmar
SRE: Skeletal-related events

## References

[1] Bray F, Ferlay J, Soerjomataram I, Siegel RL, Torre LA, Jemal A (2018). Global cancer statistics 2018: GLOBOCAN estimates of incidence and mortality worldwide for 36 cancers in 185 countries. CA Cancer J Clin.

[2] Chonprasertsuk S, Vilaichone R-K (2017). Epidemiology and treatment of hepatocellular carcinoma in Thailand. Jpn J Clin Oncol.

[3] Abbas A, Medvedev S, Shores N, Bazzano L, Dehal A, Hutchings J (2014). Epidemiology of metastatic hepatocellular carcinoma, a nationwide perspective. Dig Dis Sci.

[4] Lu Y, Hu J-G, Lin X-J, Li X-G. Bone metastases from hepatocellular carcinoma: clinical features and prognostic factors. Hepatobiliary Pancreat Dis Int HBPD INT. 2017;16:499–505. 10.1016/S1499-3872(16)60173-X 28992882

[5] Zhang D, Xu W, Liu T, Yin H, Yang X, Wu Z (2013). Surgery and prognostic factors of patients with epidural spinal cord compression caused by hepatocellular carcinoma metastases: retrospective study of 36 patients in a single center. Spine..

[6] Uei H, Tokuhashi Y (2020). Prognostic scoring system for metastatic spine tumors derived from hepatocellular carcinoma. J Orthop Surg.

[7] Kim S, Choi Y, Kwak D-W, Lee HS, Hur W-J, Baek YH (2019). Prognostic factors in hepatocellular carcinoma patients with bone metastases. Radiat Oncol J.

[8] Tomita K, Kawahara N, Kobayashi T, Yoshida A, Murakami H, Akamaru T (2001). Surgical strategy for spinal metastases. Spine..

[9] Tokuhashi Y, Uei H, Oshima M, Ajiro Y (2014). Scoring system for prediction of metastatic spine tumor prognosis. World J Orthop.

[10] Tokuhashi Y, Matsuzaki H, Oda H, Oshima M, Ryu J (2005). A revised scoring system for preoperative evaluation of metastatic spine tumor prognosis. Spine..

[11] Torre LA, Bray F, Siegel RL, Ferlay J, Lortet-Tieulent J, Jemal A (2015). Global cancer statistics, 2012. CA Cancer J Clin.

[12] Dohzono S, Sasaoka R, Takamatsu K, Nakamura H (2017). Overall survival and prognostic factors in patients with spinal metastases from lung cancer treated with and without epidermal growth factor receptor tyrosine kinase inhibitors. Int J Clin Oncol.

[13] Sciubba DM, Goodwin CR, Yurter A, Ju D, Gokaslan ZL, Fisher C (2016). A systematic review of clinical outcomes and prognostic factors for patients undergoing surgery for spinal metastases secondary to breast cancer. Glob Spine J.

[14] Tatsui CE, Suki D, Rao G, Kim SS, Salaskar A, Hatiboglu MA (2014). Factors affecting survival in 267 consecutive patients undergoing surgery for spinal metastasis from renal cell carcinoma. J Neurosurg Spine.

[15] Crnalic S, Löfvenberg R, Bergh A, Widmark A, Hildingsson C (2012). Predicting survival for surgery of metastatic spinal cord compression in prostate cancer: a new score. Spine..

[16] Sellin JN, Suki D, Harsh V, Elder BD, Fahim DK, McCutcheon IE (2015). Factors affecting survival in 43 consecutive patients after surgery for spinal metastases from thyroid carcinoma. J Neurosurg Spine..

[17] Sangsin A, Saiudom D, Pongmanee S, Saengsin J, Leerapun T, Murakami H (2018). Natural history and prognostic factors of cholangiocarcinoma with spinal metastasis: a 10-year single center study. Clin Spine Surg.

[18] Bhatia R, Ravulapati S, Befeler A, Dombrowski JJ, Gadani SD, Poddar N (2016). Hepatocellular carcinoma with bone metastases: incidence, prognostic significance and management—single center experience. J Clin Oncol.

[19] Goodwin CR, Yanamadala V, Ruiz-Valls A, Abu-Bonsrah N, Shankar G, Sankey EW (2016). A systematic review of metastatic hepatocellular carcinoma to the spine. World Neurosurg.

[20] Chang SS, Luo JC, Chao Y, Chao JY, Chi KH, Wang SS (2001). The clinical features and prognostic factors of hepatocellular carcinoma patients with spinal metastasis. Eur J Gastroenterol Hepatol.

[21] Chang U-K, Kim M-S, Han CJ, Lee DH (2014). Clinical result of stereotactic radiosurgery for spinal metastasis from hepatocellular carcinoma: comparison with conventional radiation therapy. J Neuro-Oncol.

[22] He S, Wei H, Ma Y, Zhao J, Xu W, Xiao J (2017). Outcomes of metastatic spinal cord compression secondary to primary hepatocellular carcinoma with multidisciplinary treatments. Oncotarget..

[23] Chen H, Xiao J, Yang X, Zhang F, Yuan W (2010). Preoperative scoring systems and prognostic factors for patients with spinal metastases from hepatocellular carcinoma. Spine..

[24] Rim CH, Choi C, Choi J, Seong J (2017). Establishment of a disease-specific graded prognostic assessment for hepatocellular carcinoma patients with spinal metastasis. Gut Liver.

[25] Rades D, Hansen O, Jensen LH, Dziggel L, Staackmann C, Doemer C (2019). Radiotherapy for metastatic spinal cord compression with increased radiation doses (RAMSES-01): a prospective multicenter study. BMC Cancer.

[26] Lee MH, Lee S-H, Kim E-S, Eoh W, Chung S-S, Lee C-S (2015). Survival-related factors of spinal metastasis with hepatocellular carcinoma in current surgical treatment modalities : a single institute experience. J Korean Neurosurg Soc.

[27] El-Fattah MA, Aboelmagd M, Elhamouly M (2017). Prognostic factors of hepatocellular carcinoma survival after radiofrequency ablation: a US population-based study. United Eur Gastroenterol J.

[28] Ikeda M, Okada S, Yamamoto S, Sato T, Ueno H, Okusaka T (2002). Prognostic factors in patients with hepatocellular carcinoma treated by transcatheter arterial embolization. Jpn J Clin Oncol.

[29] Gokcan H, Savaş N, Oztuna D, Moray G, Boyvat F, Haberal M (2015). Predictors of survival in hepatocellular carcinoma patients. Ann Transplant.

[30] Wang C, Li S. Clinical characteristics and prognosis of 2887 patients with hepatocellular carcinoma. Medicine (Baltimore). 2019;98. 10.1097/MD.0000000000014070. 10.1097/MD.0000000000014070 PMC6358325 30681563

[31] Carr BI, Guerra V, Giannini EG, Farinati F, Ciccarese F, Rapaccini GL, et al. A liver index and its relationship to indices of HCC aggressiveness. J Integr Oncol. 2016;5. 10.4172/2329-6771.1000178. 10.4172/2329-6771.1000178 PMC5450974 28580457

[32] Carr BI, Guerra V, Giannini EG, Farinati F, Ciccarese F, Rapaccini GL (2014). Association of abnormal plasma bilirubin with aggressive HCC phenotype. Semin Oncol.

[33] Wang X, Wang Z, Wu L. Combined measurements of tumor number and size helps estimate the outcome of resection of Barcelona clinic liver cancer stage B hepatocellular carcinoma. BMC Surg. 2016;16. 10.1186/s12893-016-0135-4. 10.1186/s12893-016-0135-4 PMC4837634 27094483

[34] Moons KGM, Altman DG, Vergouwe Y, Royston P. Prognosis and prognostic research: application and impact of prognostic models in clinical practice. BMJ. 2009;338. 10.1136/bmj.b606. 10.1136/bmj.b606 19502216

[35] Miladinovic B, Kumar A, Mhaskar R, Kim S, Schonwetter R, Djulbegovic B (2012). A flexible alternative to the cox proportional hazards model for assessing the prognostic accuracy of hospice patient survival. PLoS One.

[36] Royston P, Altman DG (2013). External validation of a cox prognostic model: principles and methods. BMC Med Res Methodol.

[37] Royston P, Parmar MKB (2002). Flexible parametric proportional-hazards and proportional-odds models for censored survival data, with application to prognostic modelling and estimation of treatment effects. Stat Med.

[38] Christakis NA, Lamont EB (2000). Extent and determinants of error in doctors’ prognoses in terminally ill patients: prospective cohort study. BMJ..

[39] Vittinghoff E, McCulloch CE (2007). Relaxing the rule of ten events per variable in logistic and cox regression. Am J Epidemiol.

[40] Moons KGM, Altman DG, Reitsma JB, Ioannidis JPA, Macaskill P, Steyerberg EW (2015). Transparent reporting of a multivariable prediction model for individual prognosis or diagnosis (TRIPOD): explanation and elaboration. Ann Intern Med.

[41] Royston P, Altman DG, Sauerbrei W (2006). Dichotomizing continuous predictors in multiple regression: a bad idea. Stat Med.

