# Supplementary material for: Natural disease progression and novel survival prediction model for hepatocellular carcinoma with spinal metastases: a 10-year single-center study
Source: World J Surg Oncol. 2020 Jun 20;18:135. doi: 10.1186/s12957-020-01913-9 (PMC7306143; doi:10.1186/s12957-020-01913-9)
Supplement: Supplementary file 1 — Additional file 1: Supplementary Table 1. Estimated log hazard ratios in the full and reduced multivariable flexible parametric regression models. Supplementary Table 2. Score chart with predicted survival probabilities at 3 months after diagnosis of spinal metastases. Supplementary Table 3. Score chart with predicted survival probabilities at 6 months after the diagnosis of spinal metastases. Supplementary Table 4. Score chart with predicted survival probabilities at 12 months after the diagnosis of spinal metastases. Supplementary Figure 1. Kaplan–Meier curves for comparative validation between the newly derived model’s prediction and the traditional Tomita and Revised Tokuhashi’s score in predicting which patients would survive more or less than 6 months. The P-values of the log-rank test are shown for each panel. [file 12957_2020_1913_MOESM1_ESM.docx]

**Supplementary Table 1.** Estimated log hazard ratios in the full and reduced multivariable flexible parametric regression models

| Predictors | Full model | | | Reduced model | | |
| --- | --- | --- | --- | --- | --- | --- |
|  | Beta | 95% CI | *P*-value | Beta | 95% CI | *P*-value |
| Age group (years) |  |  |  |  |  |  |
| ≤60 | 0.000 | Reference |  | 0.000 | Reference |  |
| >60 | 0.754 | –0.006, 1.514 | 0.052 | 0.571 | –0.029, 1.172 | 0.062 |
| Karnofskys performance status |  |  |  |  |  |  |
| Good | 0.000 | Reference |  | 0.000 | Reference |  |
| Moderate | 0.623 | –0.338, 1.585 | 0.204 | 0.692 | –0.046, 1.430 | 0.066 |
| Poor | 1.291 | 0.476, 2.105 | 0.002 | 1.085 | 0.390, 1.779 | 0.002 |
| Cirrhosis |  |  |  |  |  |  |
| No | 0.000 | Reference |  |  | Not included |  |
| Yes | 0.184 | –0.505, 0.873 | 0.600 |  |  |  |
| Ascites |  |  |  |  |  |  |
| No | 0.000 | Reference |  |  | Not included |  |
| Yes | 0.049 | –1.132, 1.229 | 0.935 |  |  |  |
| Total bilirubin (mg/dL) |  |  |  |  |  |  |
| <2.0 | 0.000 | Reference |  | 0.000 | Reference |  |
| 2.0–3.0 | 1.185 | –1.63, 2.533 | 0.085 | 0.798 | –0.193, 1.790 | 0.114 |
| >3.0 | 2.221 | 0.901, 3.541 | 0.001 | 2.346 | 1.366, 3.326 | <0.001 |
| Serum albumin (mg/dL) |  |  |  |  |  |  |
| >3.5 | 0.000 | Reference |  |  | Not included |  |
| 2.8–3.5 | –0.220 | –1.088, 0.648 | 0.619 |  |  |  |
| <2.8 | 0.978 | 0.052, 1.904 | 0.039 |  |  |  |
| Number of primary tumor |  |  |  |  |  |  |
| Single tumor | 0.000 | Reference |  | 0.000 | Reference |  |
| Multiple tumor | 1.229 | 0.206, 2.252 | 0.019 | 0.966 | 0.255, 1.677 | 0.008 |
| Portal vein involvement |  |  |  |  |  |  |
| No | 0.000 | Reference |  |  | Not included |  |
| Yes | 0.379 | –0.288, 1.046 | 0.265 |  |  |  |
| Visceral organ metastasis |  |  |  |  |  |  |
| No | 0.000 | Reference |  |  | Not included |  |
| Yes | 0.177 | –0.158, 0.512 | 0.301 |  |  |  |
| Number of vertebral columns involved |  |  |  |  |  |  |
| 1 | 0.000 | Reference |  |  | Not included |  |
| 2 | 0.093 | –0.655, 0.841 | 0.807 |  |  |  |
| ≥3 | 0.528 | –0.368, 1.425 | 0.248 |  |  |  |
| Number of extraspinal bone metastases |  |  |  |  |  |  |
| 0 | 0.000 | Reference |  |  | Not included |  |
| 1–2 | –0.076 | –1.175, 1.023 | 0.892 |  |  |  |
| ≥3 | 0.026 | –1.108, 1.159 | 0.964 |  |  |  |
| Restricted Cubic Splines |  |  |  |  |  |  |
| RCS1 | 1.866 | 1.458, 2.275 |  | 1.651 | 1.308, 1.993 |  |
| RCS2 | 0.129 | –0.159, 0.417 |  | 0.179 | –0.069, 0.427 |  |
| RCS3 | 0.000 | –0.188, 0.188 |  | 0.032 | –0.118, 0.183 |  |
| RCS4 | –0.220 | –0.346, –0.093 |  | –0.213 | –0.317, –0.110 |  |
| Model Intercept (constant) | –3.597 | –4.966, –2.227 |  | –2.492 | –3.419, –1.565 |  |

Abbreviations: CI, confidence interval; RCS, restricted cubic spline function

**Supplementary Table 2.** Score chart with predicted survival probabilities at 3 months after diagnosis of spinal metastases

| HCC-SM CMU survival  prediction model | | Prediction at 3 months | | | | | |
| --- | --- | --- | --- | --- | --- | --- | --- |
|  |  | Age ≤60 years | | | Age >60 years | | |
|  |  | Good  KPS | Moderate KPS | Poor  KPS | Good  KPS | Moderate KPS | Poor  KPS |
| Total bilirubin  <2 mg/dL | Single  tumor | **89.5 (74.9–95.9)** | **80.2 (60.2–90.8)** | **72.1 (48.5–86.3)** | **82.2 (56.2–93.6)** | **67.6 (41.0–84.2)** | **56.0 (24.9–78.6)** |
|  | Multiple  tumors | **74.8 (57.6–85.8)** | **56.0 (34.3–73.0)** | **42.3 (23.4–60.2)** | **59.8 (31.5–79.5)** | **35.8 (15.3–56.9)** | **21.8 (5.5–44.9)** |
| Total bilirubin  2–3 mg/dL | Single  tumor | **78.2 (38.3–93.9)** | **61.2 (21.0–85.7)** | **48.3 (9.5–79.9)** | **64.7 (13.4–91.0)** | **41.9 (4.8–78.0)** | **27.6 (7.8–71.1)** |
|  | Multiple  tumors | **52.4 (14.7–80.5)** | **27.5 (3.8–60.2)** | **14.8 (0.8–47.2)** | **31.9 (1.5–73.1)** | **10.2 (0.1–45.0)** | **3.4 (0–33.2)** |
| Total bilirubin >3 mg/dL | Single  tumor | **31.5 (1.2–73.8)** | **10.0 (0.1–49.0)** | **3.3 (0–35.0)** | **12.9 (0–61.5)** | **1.7 (0–28.6)** | **0.2 (0–17.7)** |
|  | Multiple  tumors | **4.8 (0–35.7)** | **0.2 (0–10.2)** | **0 (0–3.3)** | **0.5 (0–20.0)** | **0 (0–1.8)** | **0 (0–0.4)** |
| Predicted Survival Probability 81–100% 61–80% 41–60% 21–40% 0–20% | | | | | | | |

Abbreviations: KPS, Karnofsky Performance Status.

**Supplementary Table 3.** Score chart with predicted survival probabilities at 6 months after the diagnosis of spinal metastases

| HCC-SM CMU survival  prediction model | | Prediction at 6 months | | | | | |
| --- | --- | --- | --- | --- | --- | --- | --- |
|  |  | Age ≤ 60 years | | | Age > 60 years | | |
|  |  | Good  KPS | Moderate KPS | Poor  KPS | Good  KPS | Moderate KPS | Poor  KPS |
| Total bilirubin  <2 mg/dL | Single  tumor | **83.4 (62.1–93.3)** | **69.5 (43.3–85.4)** | **58.4 (30.3–78.5)** | **72.5 (38.9–89.6)** | **52.5 (23.3–75.2)** | **38.6 (10.3–67.1)** |
|  | Multiple  tumors | **62.0 (41.2–77.3)** | **38.5 (17.8–58.9)** | **24.3 (9.6–42.6)** | **42.9 (15.7–67.9)** | **18.4 (5.1–38.3)** | **8.2 (1.0–25.8)** |
| Total bilirubin  2–3 mg/dL | Single  tumor | **66.7 (19.8–90.4)** | **44.6 (7.1–78.2)** | **30.2 (1.8–69.9)** | **48.9 (3.4–85.9)** | **23.9 (0.6–67.1)** | **12.0 (0–57.8)** |
|  | Multiple  tumors | **34.6 (4.0–70.4)** | **12.0 (0.4–44.2)** | **4.3 (0–30.0)** | **15.2 (0.1–60.1)** | **2.3 (0–27.3)** | **3.8 (0.4–16.8)** |
| Total bilirubin >3 mg/dL | Single  tumor | **15.0 (0.1–62.2)** | **2.2 (0–33.0)** | **0.4 (0–19.6)** | **3.5 (0–46.4)** | **0.1 (0–14.1)** | **0 (0–6.6)** |
|  | Multiple  tumors | **0.7 (0–19.9)** | **0 (0–2.9)** | **0 (0–0.5)** | **0 (0–7.7)** | **0 (0–0.2)** | **0 (0)** |
| Predicted Survival Probability 81–100% 61–80% 41–60% 21–40% 0–20% | | | | | | | |

Abbreviations: KPS, Karnofsky Performance Status.

**Supplementary Table 4.** Score chart with predicted survival probabilities at 12 months after the diagnosis of spinal metastases

| HCC-SM CMU survival  prediction model | | Prediction at 12 months | | | | | |
| --- | --- | --- | --- | --- | --- | --- | --- |
|  |  | Age ≤ 60 years | | | Age > 60 years | | |
|  |  | Good  KPS | Moderate KPS | Poor  KPS | Good  KPS | Moderate KPS | Poor  KPS |
| Total bilirubin  <2 mg/dL | Single  tumor | **69.4 (43.1**–**85.4)** | **48.3 (21.6**–**70.7)** | **34.0 (11.2**–**58.8)** | **52.4 (17.8**–**78.5)** | **27.5 (6.5**–**54.4)** | **14.8 (1.3**–**42.9)** |
|  | Multiple  tumors | **38.3 (19.4**–**57.1)** | **14.7 (3.2**–**34.4)** | **5.9 (0.9**–**18.0)** | **18.3 (2.8**–**44.7)** | **3.4 (0.2**–**15.3)** | **0.7 (0**–**7.1)** |
| Total bilirubin  2–3 mg/dL | Single  tumor | **44.5 (4.7**–**80.7)** | **19.8 (0.6–60.0)** | **9.1 (0–47.8)** | **23.8 (0.2–72.8)** | **5.7 (0–44.4)** | **1.4 (0–32.9)** |
|  | Multiple  tumors | **11.9 (0.2**–**49.1)** | **1.4 (0**–**20.3)** | **0.2 (0–9.7)** | **2.3 (0–36.1)** | **0 (0–8.3)** | **0 (0–3.2)** |
| Total bilirubin >3 mg/dL | Single  tumor | **2.2 (0**–**35.9)** | **0 (0–9.7)** | **0 (0–3.3)** | **0.1 (0–19.7)** | **0 (0–1.7)** | **0 (0–0.4)** |
|  | Multiple  tumors | **0 (0**–**3.6)** | **0 (0–0.1)** | **0 (0)** | **0 (0–0.6)** | **0 (0)** | **0 (0)** |
| Predicted Survival Probability 81–100% 61–80% 41–60% 21–40% 0–20% | | | | | | | |

Abbreviation: KPS, Karnofsky Performance Status.


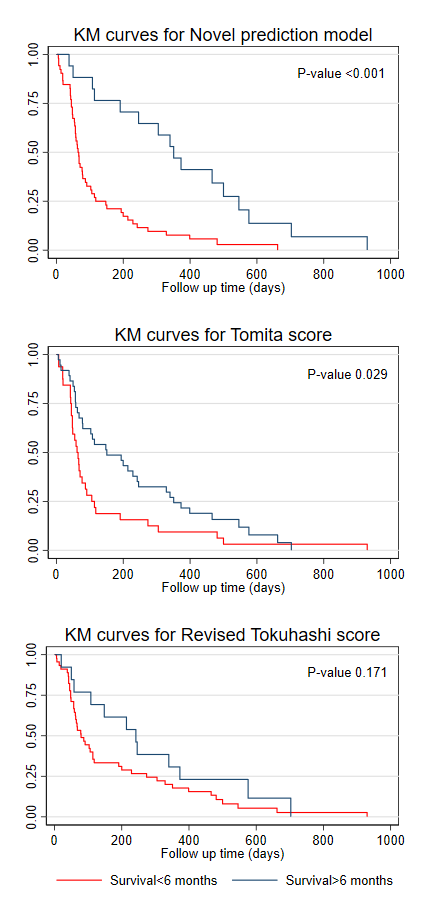


**Supplementary Figure 1.** Kaplan–Meier curves for comparative validation between the newly derived model’s prediction and the traditional Tomita and Revised Tokuhashi’s score in predicting which patients would survive more or less than 6 months. The *P*-values of the log-rank test are shown for each panel.
